# Depressive Symptoms, HIV Medication Adherence, and HIV Clinical Outcomes in Tanzania: A Prospective, Observational Study

**DOI:** 10.1371/journal.pone.0095469

**Published:** 2014-05-05

**Authors:** Nadya M. Belenky, Stephen R. Cole, Brian W. Pence, Dafrosa Itemba, Venance Maro, Kathryn Whetten

**Affiliations:** 1 Department of Epidemiology, University of North Carolina Gillings School of Global Public Health, Chapel Hill, North Carolina, United States of America; 2 Center for Health Policy & Inequalities Research, Duke Global Health Institute, Department of Community and Family Medicine, Duke University, Durham, North Carolina, United States of America; 3 Tanzania Women Research Foundation, Moshi, Tanzania; 4 Kilimanjaro Christian Medical Center, Moshi, Tanzania; University of Pittsburgh Center for Vaccine Research, United States of America

## Abstract

Depressive symptoms have been shown to independently affect both antiretroviral therapy (ART) adherence and HIV clinical outcomes in high-income countries. We examined the prospective relationship between depressive symptoms and adherence, virologic failure, and suppressed immune function in people living with HIV/AIDS in Tanzania. Data from 403 study participants who were on stable ART and engaged in HIV clinical care were analyzed. We assessed crude and adjusted associations of depressive symptoms and ART adherence, both at baseline and at 12 months, using logistic regression. We used logistic generalized estimating equations to assess the association and 95% confidence intervals (CI) between depressive symptoms and both virologic failure and suppressed immune function. Ten percent of participants reported moderate or severe depressive symptoms at baseline and 31% of participants experienced virologic failure (>150 copies/ml) over two years. Depressive symptoms were associated with greater odds of reported medication nonadherence at both baseline (Odds Ratio [OR] per 1-unit increase  = 1.18, 95% CI [1.12, 1.24]) and 12 months (OR  = 1.08, 95% CI [1.03, 1.14]). By contrast, increases in depressive symptom score were inversely related to both virologic failure (OR = 0.93, 95% CI [0.87, 1.00]) and immune system suppression (OR = 0.88, 95% CI [0.79, 0.99]), though the association between depressive symptoms and clinical outcomes was less precise than for the association with nonadherence. Findings indicate a positive association between depressive symptoms and nonadherence, and also an inverse relationship between depressive symptoms and clinical outcomes, possibly due to informative loss to follow-up.

## Introduction

Clinical outcomes of HIV have dramatically improved since the widespread adoption of modern combination antiretroviral therapy (ART), which has enhanced quality of life and increased survival time among people living with HIV/AIDS (PLWHA) [1], [2]. However, despite ART's success, PLWHA who are on ART still exhibit variability in adherence and clinical outcomes [3]. This variability has been partially attributed to behavioral and psychosocial factors, including depression [4]–[8].

The effects of the mental health disease burden in PLWHA may be exacerbated by lack of treatment options. The World Health Organization's most recent estimates from the Mental Health Gap Action Programme indicate that 75% of those in need of mental health treatment do not have access to services [9]. Furthermore, despite similar prevalence of mental health disorders, mental health resources vary widely by country; in low-income countries, the World Health Organization calculated a median number of 0.3 mental health professionals per 100,000, compared to a median number of 70 per 100,000 in high-income countries [10].

Depressive symptoms are thought to influence treatment initiation and maintenance of PLWHA through different pathways, from immunological response to health-related behaviors. Studies have shown that depressive symptoms predict differences in HIV disease progression, even after controlling for ART adherence [8]. Depressive symptoms have also been linked to general suppressed immune function [11], increased HIV-1 viral load (VL) [12], faster progression to AIDS [4], as well as higher all-cause and HIV-related mortality [13]–[16]. Depressive symptoms also affect health-related behaviors and have been linked to illicit drug use, alcohol consumption, and reduced ART adherence [17], [18].

The effects of depressive symptoms are also not restricted to high-income countries. A recent systematic review on ART adherence in sub-Saharan Africa [19] estimated the prevalence of depressive symptoms was 31%, pooled over 23 studies and 9 countries, and that the likelihood of achieving good ART adherence was 55% lower among PLWHA with depressive symptoms compared to those without. However, 20 of the 23 studies on depressive symptoms were cross-sectional, most consisted of individuals with advanced HIV, and access to care varied widely.

Previous studies have examined the independent associations of depressive symptoms and HIV clinical outcomes. To date, however, no study has considered the relationship of depressive symptoms with ART adherence and HIV clinical outcomes at separate time points in a sub-Saharan population in established medical care.

Using data from the Coping with HIV/AIDS in Tanzania (CHAT) study, we quantify the association of baseline depressive symptoms on ART adherence at baseline and 12-months as well as examine the relationship between baseline depressive symptoms and both virologic failure and suppressed immune function at any point during follow-up (i.e., 12, 24, or 36 months). We hypothesize that participants with high depressive symptom scores are more likely to report nonadherence than those with low depressive symptom scores, and that participants with high depressive symptom scores are more likely to experience virologic failure and suppressed immune function. The results of this study will add to the limited body of knowledge on the relationship between depressive symptoms on both ART adherence and HIV clinical outcomes in low- and middle-income countries.

## Methods

### Participants

The CHAT Study is a prospective observational study that examines the relationship between psychosocial characteristics, ART adherence, and HIV health outcomes. The study recruited 1,191 participants in Moshi, Tanzania, between November 2008 and October 2009. Study participants form five cohorts: patients with established HIV infection receiving care at a tertiary referral hospital (n = 227), patients with established HIV infection receiving care at a public hospital (n = 271), individuals recently diagnosed with HIV (n = 263) or tested negative for HIV (n = 181) at voluntary counseling and testing sites, and a random community sample of adults (n = 249). Sampling, recruitment, and selection procedures are described in more detail elsewhere [20]. All participants complete standardized in-person interviews in Swahili every six months. In addition to measurements obtained during the clinical exam at study initiation, participants with established HIV infection also provide blood samples for VL during a clinical exam every 12 months and corresponding CD4 cell counts are abstracted from medical records.

We restricted this analysis to participants with established HIV infection (i.e., who were either receiving care at a tertiary referral hospital or a public hospital, n = 498). We further restricted the sample to participants who were on ART at baseline (n = 403). Written, informed consent was obtained from all participants. The study was approved by the Kilimanjaro Christian Medical Center Institutional Review Board in Tanzania and the Duke University Health System Institutional Review Board in the United States.

### Measurements

Age, gender, marital status (i.e., single, married, divorced/widowed), education (i.e., none, primary, secondary, university), and household assets were assessed at baseline using the in-person questionnaire. Possible household assets included access to: running water, a flush toilet, and/or a television; each asset contributed one point to the household asset score (range: 0–3).

Depressive symptoms were measured using the Patient Health Questionnaire (PHQ-9), a nine-item questionnaire taken from the Primary Care Evaluation of Mental Disorders [21], [22], which has been validated in similar populations [23], [24]. For each of the nine items, participants were asked how often they experienced the corresponding symptom during the previous two weeks. Individual items are scored on a 0–3 scale. Total scores for depressive symptoms range from 0–27, where higher scores indicate greater depressive symptom severity. In this analysis, depressive symptoms at baseline were examined continuously.

The outcomes of this study were ART nonadherence at baseline and 12 months, ever having experienced a virologic failure, or ever having had suppressed immune function. ART nonadherence was assessed using a combination of four ART adherence questions. Answering affirmatively that a dose had been missed within the last month or indicating either that doses were missed, forgotten about, or skipped; not all doses were taken; or that doses were taken more than one hour early or late. All indications of missed or mistimed doses were assessed using a visual guide. The definition of ART nonadherence included dose timing based on previous findings that dose timing error was significantly related to HIV VL [25].

Plasma for VL measurements was collected from participants in 10-ml Vacutainer tubes containing potassium ethylenediaminetetraacetic acid and was stored at −80°C until testing. VLs were quantified by HIV-1 RNA extracted from 1.0-ml plasma samples using an isothermal nucleic acid sequence-based amplification method. This assay has a lower detection limit of 40 or 150 copies/ml depending on the lowest sample volume of the run, and is linear to concentrations as high as 10^6^ copies/ml. An Abbott m2000 system was used to analyze all samples and the study laboratory participates in an international viral quality assurance program. Initial CD4 cell counts were obtained during the clinical exam at study entry. During follow-up, the CD4 cell counts that corresponded in time to the VL measurements were abstracted from medical charts.

### Statistical Analyses

For all outcomes, we adjusted for baseline measures of age, gender, marital status, education, and asset score. We estimated the adjusted and unadjusted association between depressive symptoms and ART adherence (at baseline and 12 months), and virologic failure during follow-up or suppressed immune function at any available measurement during follow-up. Odds ratios (OR) for ART adherence were obtained using logistic regression. Generalized estimating equation (GEE) logistic models were used to account for repeated measurements of viral load and CD4 cell counts. Virologic failure was examined as a dichotomous variable, where virologic failure was defined as a viral load of >150 copies/ml at any point after baseline. Suppressed immune function was also treated as a dichotomous variable, where suppressed immune function was defined as any instance of a CD4 cell count below 200 cells/mm^3^ after baseline. Both GEE logistic models of virologic failure and suppressed immune function were also examined after adjusting for baseline values of viral load and CD4 cell count, respectively. All statistical analyses were performed using STATA version 12.1 for Mac (Stata Corporation, College Station, Texas).

## Results

Of the 403 participants, 69% (n = 276) of the participants were female and the median age was 42 years (IQR: 36–48). Approximately 37% of the study participants were married or co-habitating, 14% had never married, 28% were widowed, and 20% were divorced. Seventy-four percent (n = 297) of participants had received a primary education and the median asset score was 2, corresponding to ownership of two of the three measured assets: running water, a flush toilet, or a television.

Levels of depressive symptoms were low: only 10% (n = 40) of participants had a score indicating moderate or severe depressive symptoms (PHQ-9≥10) and 23% (n = 94) had scores indicating of mild, moderate, or severe depressive symptoms (PHQ-9≥5). At baseline, the median CD4 count was 305 cells/mm^3^ (IQR: 191–443 cells/mm^3^). The majority of participants had a suppressed VL at baseline (82%), where suppression was defined as a VL of ≤150 copies/ml (Table 1).

**Table 1:** Baseline characteristics of 403 CHAT study participants, Tanzania, 2008–2009.

Characteristic^as^		Median (IQR) or n (%)
Sample Size		403
Age		42 (36–48)
Female gender, n (%)		276 (68.5)
Marital status, n (%)	Married or cohabitating	149 (37.0)
	Never married	57(14.1)
	Widowed	112 (27.8)
	Divorced	80 (19.9)
Education, n (%)	None	21 (5.2)
	Primary	297 (73.7)
	Secondary	70 (17.4)
	University	10 (2.5)
Household assets^b^, n (%)	Zero	46 (11.6)
	One	62 (15.6)
	Two	103 (25.9)
	Three	187 (47.0)
Suppressed immune function, n (%) (<200 CD4 cells/mm3)		76 (18.9)
Virologic failure, n (%)		72 (17.9)
Depressive symptoms^c^, n (%)	None or mild	358 (88.8)
	Moderate or severe	40 (9.9)
Nonadherent, n (%)		94 (23.3)

a Median (IQR) unless otherwise noted.

b Household asset score was determined by access to: running water, a flush toilet, and/or a television.

c Depressive symptoms are measured using the Patient Health Questionnaire (range: 0–27).

VL measurements at baseline were available for 375 participants (93%) and a high percentage of participants had VL measurements at 12, 24 and 36 months (84%, 86%, and 91%, respectively). The proportion of participants experiencing virologic failure changed over time, ranging from 10% to 19%, and 31% of participants experienced at least one virologic failure. CD4 cell counts were based on the study's medical exam at the initial study visit and abstracted from medical records during follow-up. At baseline, 285 (71%) of 403 participants had CD4 cell count measurements. During follow-up, the number of participants with CD4 cell count measurements was lower, where the percentage of participants with CD4 cell counts ranged from 42% to 56%. Overall, 97% of participants had at least one VL measurement and 84% had at least one CD4 cell count measurement over the time points used in this analysis (Table 2).

**Table 2:** Virologic failure and immune suppression over study visits.

Outcome Measure	Baseline	12 months	24 months	36 months	Overall
Virologic Failure, x/n^a^ (%)	72/375 (19.2)	35/340 (10.3)	39/348 (11.2)	53/366 (14.5)	120/392 (30.6)
CD4 <200, x/n^b^ (%)	76/285 (26.7)	34/212 (16.0)	22/227 (9.7)	13/170 (7.7)	98/381 (25.7)

a Number of virologic failures is indicated by x, number of available VL measurements are indicated by n.

b Number of CD4 counts defining suppressed immune function indicated by x, number of available CD4 counts indicated by n.

Results show a positive association between depressive symptoms and ART nonadherence both at baseline and at 12 months. The unadjusted estimates of the relationship between baseline depressive symptoms and ART nonadherence at baseline and 12 months ART were OR = 1.16 (95% CI: 1.10, 1.22) and 1.08 (95% CI: 1.03, 1.14), respectively. After adjustment for potential confounders (age, gender, marital status, education, and household assets), the magnitude of the relationship was virtually unchanged, resulting in an OR of 1.18 (95% CI: 1.12–1.24) and 1.08 (95% CI: 1.03, 1.14) for ART nonadherence at baseline and 12 months, respectively. In other words, for every 1-unit increase in PHQ-9 score, the odds of being nonadherent to ART were 1.1 times greater than the odds of being adherent. The adjusted ORs for the effect of continuous depressive symptoms on virologic failure and suppressed immune function show an inverse relationship with depressive symptoms, where a higher depressive symptom score corresponds to lower odds of virologic failure or immunosuppression (OR = 0.93 [95% CI: 0.87–1.00] and OR = 0.88 [95% CI: 0.79, 0.99], respectively) (Table 3).

**Table 3:** Effect of depressive symptoms on nonadherence and clinical outcomes.

Outcome Measure	Crude OR (95% CI)	Adjusted OR^a^ (95% CI)
ART Nonadherence^b^ (Baseline)	1.16 (1.10, 1.22)	1.18 (1.12, 1.24)
ART Nonadherence^b^ (12 months)	1.08 (1.03, 1.14)	1.08 (1.03, 1.14)
Virologic Failure^c^ (VL>150 copies/ml)	0.95 (0.89, 1.01)	0.93 (0.87, 1.00)
Suppressed Immune Function^c^ (CD4<200 cells/mm^3^)	0.95 (0.86, 1.07)	0.88 (0.79, 0.99)

a Adjusted for baseline measurement of age, gender, marital status, education, and household assets.

b Any self-reported deviation from perfect ART adherence was used as indicator for nonadherence.

c Failure/suppression at any point after baseline measurement, adjusted for baseline measure of failure/suppression.

## Discussion

In this population of Tanzanian adults with HIV in established medical care, we found that a continuous measure of baseline depressive symptoms was positively associated with ART nonadherence, both at baseline and the 12-month follow-up visit. This positive relationship held in both the crude and adjusted results. Increases in baseline depressive symptoms were not associated with increased odds of either virologic failure or suppressed immune function during follow-up.

These findings are consistent with the proposed hypothesis that depressive symptoms are positively associated with ART nonadherence. Furthermore, association held in both direction and magnitude after adjustment for potential confounders. While results indicated a positive association between depressive symptoms and ART nonadherence, they also indicate an inverse relationship between depressive symptoms and clinical outcomes, possibly due to informative loss to follow-up.

Because previous studies of depressive symptoms and HIV clinical outcomes have relied on cross-sectional study designs [19], factors previously associated with ART adherence, VL or CD4 counts may not predict change over prospective follow-up. Furthermore, this analysis considered individuals in established HIV clinical care, who had been on ART for at least six months. In an ART-stabilized population, psychosocial factors may play a more important role in determining ART adherence patterns as well as early virologic suppression and maintenance. To our knowledge, this is the first study to examine the relationship between depressive symptoms, and a combination of ART adherence and clinical outcomes using a prospective design on a sub-Saharan population of PLWHA in established medical care.

Prospective assessment of ART adherence, CD4 cell counts, and VL is a primary advantage of this study, resulting in stronger causal interpretation of results due to established temporal order between exposure and outcome. Furthermore, the CHAT study's emphasis on psychosocial factors is less common in low-income countries, despite the combination of high prevalence and reduced access to mental health services. Although this study is conducted using a Tanzanian cohort, high ART adherence and retention rates increase the generalizability of findings to other populations that have similar ART adherence patterns and access to treatment. Another advantage of the CHAT Study is the measurement of depressive symptoms by the PHQ-9, which has been previously validated specifically in PLWHA in sub-Saharan Africa [23], [24]. Finally, the high retention in the cohort over the two year study period, for both psychosocial and clinic visits, makes bias due to drop-out less likely.

The analysis was limited by several factors. Levels of depressive symptoms were low in the study sample, resulting in small numbers of participants with elevated depressive symptoms and high VL or suppressed immune function. In addition, the CHAT Study is ongoing and not all clinical measurements were available at the later time points. Depressive symptoms and ART adherence measures were limited in that both are self-reported, although this is common in studies of ART adherence and depressive symptoms in sub-Saharan Africa [19]. Finally, while the PHQ-9 is an effective screening tool for depressive symptoms, it does not represent a clinical diagnosis.

This study adds to the literature on the influence of psychosocial factors on ART adherence and HIV clinical outcomes. Previous studies on psychosocial factors and ART adherence have suggested that strategies for intervention directed at depressive symptoms would have positive effects on ART adherence. This study strengthens those results for a sub-Saharan population receiving stable medical care. Furthermore, this study demonstrates that, despite the positive association between baseline depressive symptoms and ART nonadherence at baseline and 12 months, findings show an inverse relationship between depressive symptoms at baseline and odds of both virologic failure and suppressed immune function during follow-up. The confidence intervals for the clinical measures were larger than the confidence intervals for nonadherence and it is possible that the inverse association is due to incomplete clinical data and subject to change as more clinical data are incorporated. Longer periods of ART nonadherence associated with depressive symptoms may also be necessary before changes in VL or CD4 are measurable.

This analysis demonstrates an under-utilized avenue for identifying ART nonadherence in early treatment stages and maximizing the impact of HIV treatment. It also emphasizes the far-reaching impact that increased capacity for mental health screening and services can have. The association between depressive symptoms and nonadherence in the early stages of treatment indicates the advantage of screening for depressive symptoms at treatment initiation. Additional research should explore the effects of combining mental health screening and treatment with conventional HIV care on retention in clinical care, as well as the effects of mental health services in the later stages of HIV disease progression. The results of this study demonstrate the advantage of combining conventional HIV care with mental health screening for improving medication adherence and that, even in low- and middle-income settings, identification and treatment of depressive symptoms should be a priority for PLWHA.
